# Effects of Filler Wires on the Microstructure and Mechanical Properties of 2195-T6 Al-Li Alloy Spray Formed by TIG Welding

**DOI:** 10.3390/ma12213559

**Published:** 2019-10-30

**Authors:** Yuhui Zhang, Huan Li, Chuanguang Luo, Lijun Yang

**Affiliations:** 1 Tianjin Key Laboratory of Advanced Joining Technology, Tianjin University, Tianjin 300072, China; yuhui9256@163.com (Y.Z.); lihuan@tju.edu.cn (H.L.); chg_luo@163.com (C.L.); 2 Sichuan Institution of Aerospace Systems Engineering, Chengdu 610100, China

**Keywords:** aluminum-lithium alloy, TIG welding, microstructure, tensile strength, fracture morphology

## Abstract

The main purpose of this work was to investigate the microstructure and mechanical properties of spray-formed 2195-T6 Al-Li alloy welding joints produced by tungsten inert gas (TIG) with Al-Cu and Al-Si-Cu filler wires, so that they can be better used in space vehicle tanks. The porosity analysis indicates that the porosity area of the weld seam with the Al-Si-Cu filler wire is approximately 7.989 times larger than that of the Al-Cu filler wire. Furthermore, the microstructure and microhardness results indicate that the Al/Cu eutectic near the fusion line distributes more at the grain boundaries, while more dispersed Al_2_Cu phase is found inside the grain, which improves the strength of the joint when using Al-Cu filler wire. However, when using the Al-Si-Cu filler wire, more Si, Cu, and Ti elements are segregated at the grain boundaries, forming a brittle-hard network Al/Cu/Ti eutectic, which reduces the performance of the joint. Additionally, the tensile strength and elongation of the weld joint are about 68.6% and 89.9% of the base metal (BM) when using the Al-Cu filler wire, and can approach the level of friction stir welding (FSW). However, the tensile strength and elongation are only about 56.8% and 39.9%, respectively, of the BM in the weld joint when using the Al-Si-Cu filler wire. Lastly, the fractures both occur on the fusion line and the fracture morphology of the weld joint shows that it is a mixed fracture mode dominated by plastic fracture when using Al-Cu filler wire, while it is mainly a quasi-cleavage fracture mode when using Al-Si-Cu filler wire. Therefore, the joint strength when using Al-Si-Cu filler wire with high strength matching is not as good as that of Al-Cu filler wire with low strength matching.

## 1. Introduction

With the development of the aerospace industry, the requirements for light-weight and high-strength construction are getting higher and higher, so as to increase the payload and fuel efficiency. Rioja, R. J. has shown that when 1% of Li element is added to a pure aluminum alloy, the density will reduce by 3% and the elastic modulus will increase by 6% [1]. If an Al-Li alloy is used as the structure, its weight can be reduced by 10%~15% compared with other aluminum alloys [2]. 

The 2195 Al-Li alloy belongs to the third generation of Al-Li alloys. It has a high specific strength, low density, fatigue resistance, excellent low temperature performance, and corrosion resistance, which are widely used in the propellant tank of rockets and space shuttles [3,4,5]. By adjusting the ratio of Cu to Li in the alloy, a large number of finely dispersed T1(Al_2_CuLi) and θ’(Al_2_Cu) phases can be obtained, and the strength of the aluminum alloy can be improved [3].

By means of previous research, the performance of cast Al-Li alloys has been greatly improved [6,7]. However, since the alloy in the cast state exhibits a large amount of segregation, this limits the further improvement of its performance [8,9]. The sprayed alloy obtained by combining the excellent characteristics of powder metallurgy and casting has the advantages of fine crystal grains, small macro-segregation, precise control of the alloy composition, and a lower production cost. Moreover, it has been successfully applied to the 7 series aluminum alloy [10,11].

Welding is a vital process for joining of an Al-Li alloy. In recent years, electron beam welding (EBW), friction stir welding (FSW), and laser beam welding (LBW) have represented a large proportion of the welding research of Al-Li alloys. WANG Shaogang et al. [12] studied the microstructures and mechanical properties of Al-Cu-Li alloy joints welded by EBW. It was found that the strength coefficient of joints is increased from 0.64 in the welded condition to 0.90 after post-weld double aging treatment. Wang Liwei et al. [13] successfully welded the 2198 Al-Li alloy by using an ultrahigh-frequency pulse alternating current with a cold metal transfer method. It was found that the joint strength is the highest when the frequency reaches 60 kHz. Zhang J et al. [14] found that the tensile strength reaches only 65% of the base metal with the different welding parameters when using the FSW method. Furthermore, Zhang Xinyi et al. [15] investigated the LBW of the AA2060 Al-Li alloy with AlSi_12_ filler wire, and was found that the tensile strength of the weld joint was 83% of the base metal. 

Although FSW or LBW can be used to increase the strength of Al-Li alloys after welding, the equipment required is more complicated, and it is more difficult to implement for large-diameter launch vehicle tanks, such as combination welding of a melon flap and flange at the bottom of the tank. The tungsten inert gas (TIG) welding method requires a smaller amount of equipment and is flexible and convenient to operate. As Solórzano et al. [16] demonstrated, the joint ultimate strength of the TIG-welded Al-Li alloy 2091 without filler wire was about 63% of that of the base metal. Chen and Chaturvedi [4] proposed a double-V-groove of the TIG-welded casted Al-Li alloy 2195 with 4043 Al-Si filler wire, and showed that the joint ultimate strength and ductility were approximately 51.6% and 16.4% of the base metal, which still needs to be improved.

Through an analysis of the current welding situation of the Al-Li alloy, it has been found that the joint strength obtained by the common fusion welding method is not as high as LBW, FSW, and EBW. However, the TIG welding plays an important role in aerospace industrial production. Therefore, in order to improve the strength of weld joint, we chose two kinds of filler wires to control the microstructure and properties of the welded joint. One filler wire (Al-Cu) exhibits low strength matching to the base metal, while the other filler wire (Al-Si-Cu) displays high strength matching to the base metal.

In this study, the microstructure and mechanical properties of 2195 Al-Li alloy welding joints were investigated by using self-made Al-Cu and Al-Si-Cu filler wires. Firstly, TIG welding of the sprayed 2195-T6 Al-Li alloy was carried out. Tensile tests and a series of characterization tests were carried out on the samples after welding. Then, the effect of wire composition on the microstructure and properties of the sprayed 2195 Al-Li alloy was analyzed. The results show that the strength and elongation approach the level of FSW when using the Al-Cu filler wire.

## 2. Materials and Method

### 2.1. Materials 

The base metal (BM) used in this study was a spray-formed Al-Li alloy 2195-T6 plate measuring 290 mm (length) × 85 mm (width) × 6mm (thickness), which was composed of 3.66% Cu, 0.88% Li, 0.45% Mg, 0.31% Ag, 0.12% Zr, 0.067% Si, 0.036% Fe, and 0.0006% Mn. Spray-formed 2195 Al-Li alloy was in a rolling state before welding, and BM grains were distributed in a strip shape along the rolling direction. Meanwhile, there were many precipitated strengthening phases on the matrix by means of solid solution and aging treatment [11]. The filler wires for the TIG welding were Al-Cu and Al-Si-Cu, with a diameter of 3.2 mm. Table 1 lists the chemical compositions of filler wires. 

### 2.2. TIG Welding Process

**Figure 1:**
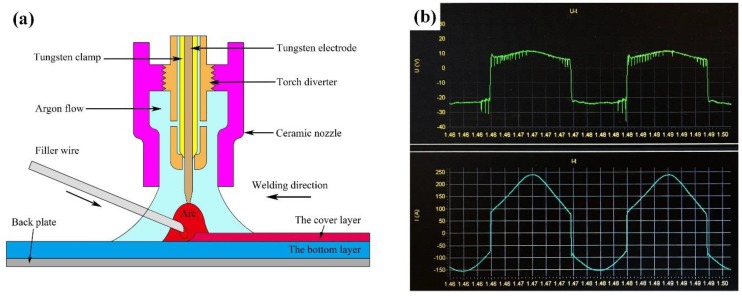
Schematic diagram of tungsten inert gas (TIG) welding (**a**) and U/I waveform (**b**).

A welding power (Lincoln Precision TIG 375, Lincoln Electric, Cleveland, OH, U.S.A) with a square wave alternate current was used for the butt-welding of sprayed 2195-T6 plates with Al-Cu and Al-Si-Cu filler wires. Prior to welding, some V-groove butt joints with an included angle of 90° and a blunt edge of 1.5 mm were employed, and the BM and filler wires were then mechanically polished and cleaned with acetone, before finally being fixed on the fixture. The average welding parameters were made by manual TIG welding, as shown in Table 2. The welding process can be divided into two passes, namely the bottom layer and top layer. The schematic diagrams of TIG welding and the voltage and current (U/I) waveform are shown in Figure 1. The time proportion of the negative polarity half cycle and positive polarity half cycle were about 40% and 60%, respectively, which effectively resulted in cathode cleaning and large penetration. In addition, for the sake of obtaining a better welding quality, the ambient temperature and humidity were set at 29 °C and 42%, respectively.

### 2.3. Microstructure and Mechanical Properties Test

After welding, the cross sections were made perpendicular and parallel to the weld. Then, samples were ground with various grit papers. Following that, the samples were polished with 1.5 μm diamond agent. The cross sections were then etched using Keller’s reagent, in which they were immersed for 18 s. Microstructures of the welding joints were examined by optical microscopy (Zeiss Vert. A1, Carl Zeiss AG, Oberkochen, Germany), ultra-depth of field microscopy (Zeiss Smart Zooms 5, Carl Zeiss AG, Oberkochen, Germany), and scanning electron microscopy (SEM, JSM-7800F, Japan Electronics Co., Ltd., Tokyo, Japan) equipped with energy dispersive spectroscopy (EDS, EDAX lnc., Philadelphia, PA, U.S.A).

**Figure 2:**
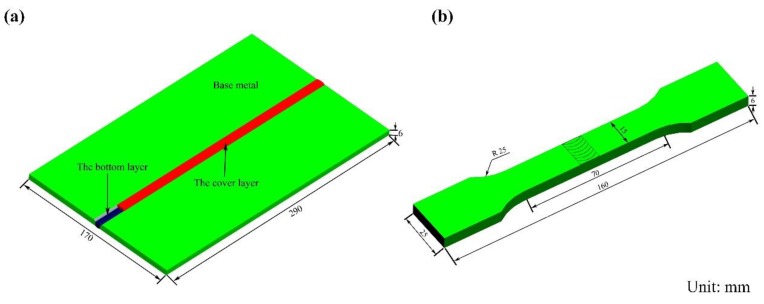
Schematic diagrams of the 3D dimensions of base metal (BM) (**a**) and tensile specimens (**b**).

The mechanical properties of welding joints were examined, including the microhardness and tensile strengths. The hardness was measured by Vickers hardness tester (Huayin HV-1000A, Laizhou Huayin Test Instrument Co., Ltd., Yantai, China) with a 9.8 N load for 10 s. The measurements were taken along three lines, which were located above, in the middle, and below the cross section, with an interval of 0.5 mm between two points. The 3D dimensions of the BM and tensile samples are illustrated in Figure 2, according to the GB/T 228.1-2010 [17] and GB/T 2651-2008 standards [18]. The tensile samples were made by wire cut electrical discharge machining (DK7735) and were examined by a tensile test machine (ddl-300) with a gauge length of 54 mm. 

## 3. Results

### 3.1. Porosity Characteristics

**Figure 3:**
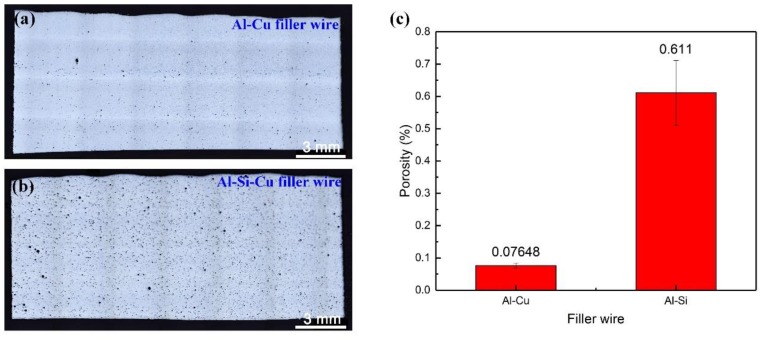
Longitudinal sections of the 2195-T6 weld seam using Al-Cu (**a**) and Al-Si-Cu (**b**) filler wires and the porosity with different filler wires (**c**).

The porosity of longitudinal sections was measured by Image J software (ImageJ 1.52a, National Institutes of Health, Bethesda, MD, U.S.A). Firstly, binary treatment was carried out on the metallographic picture to highlight the porosities and remove some small black spots. Then, based on the threshold (0.001 mm^2^) segmentation, the area of porosities on the section was determined, and finally, the proportion of the porosity area to the section was calculated, as shown in Figure 3. The porosities were densely and evenly distributed in the weld when using Al-Si-Cu and Al-Cu filler wire. The porosity area of the weld seam with the Al-Cu filler wire was approximately 7.989 times smaller than that of the Al-Si-Cu filler wire. 

**Figure 4:**
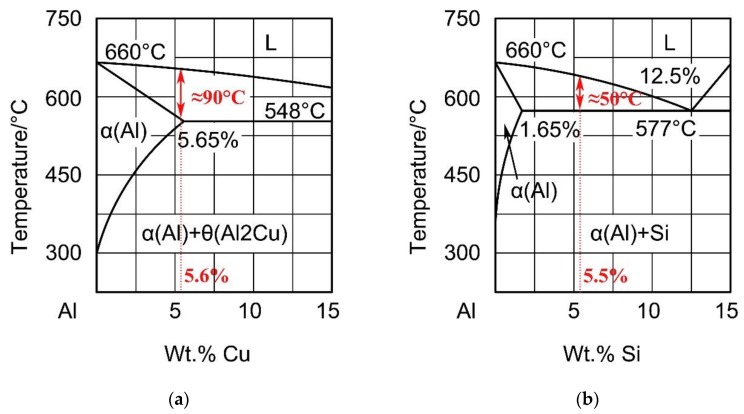
Partial phase diagrams of Al-Cu (**a**) and Al-Si (**b**).

Figure 4 shows the phase diagrams of Al-Cu and Al-Si alloys. When the content of Cu or Si are 5.6% and 5.5% in the weld seam, the range of the crystallization temperature is approximately 90 °C and 50 ℃, respectively. Furthermore, in the cooling process，the liquid phase and solid phase coexist simultaneously, under the same cooling rate, and the weld with a high Si content solidifies first. Besides, a high alloy content increased the viscosity of the liquid metal, which is not conducive to molten pool flow, and induced gas not easy to spill over in a short time. What is more, reticulated eutectic and intermetallic compound grain boundaries were composed of Al, Si, Cu, Fe, and Ti, which hinder liquid metal flow during solidification [19]. Therefore, the porosities in the weld seam with Al-Si-Cu filler wire were larger. 

As He et al. [20] discussed, porosities were the major bearing parts affecting the strength and ductility, and local deformation was concentrated around the porosities. In this case, the welding joint with an Al-Si-Cu filler wire had a lower tensile strength. 

### 3.2. Tensile Properties

**Figure 5:**
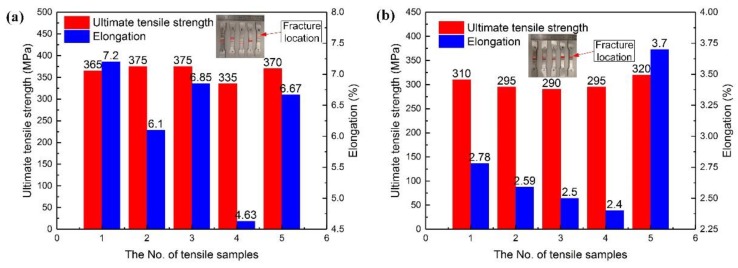
Tensile strength and elongation of welding joints using Al-Cu (**a**) and Al-Si-Cu (**b**) filler wires.

Figure 5 shows the joint strength and elongation of the plates manufactured by TIG welding with Al-Cu and Al-Si-Cu filler wire. The average strength and elongation of the joint with Al-Cu filler wire were 365 MPa and 6.29%, while those of the joint with Al-Si-Cu filler wire were 302 MPa and 2.79%, respectively. Besides, the average strength and elongation of the BM were 532 MPa and 7.0%, respectively. That is to say, the joint strength and elongation were 68.6% and 89.9% of the BM with the Al-Cu filler wire, while the joint strength and elongation were 56.8% and 39.9% of the BM with the Al-Si-Cu filler wire, respectively. In addition, most of the specimens obtained with Al-Cu filler wire were broken at the fusion line, while all the specimens obtained with Al-Si-Cu filler wire were broken at the fusion line. Therefore, the microstructure and properties near the fusion line were tested in this study.

### 3.3. Microstructure

**Figure 6:**
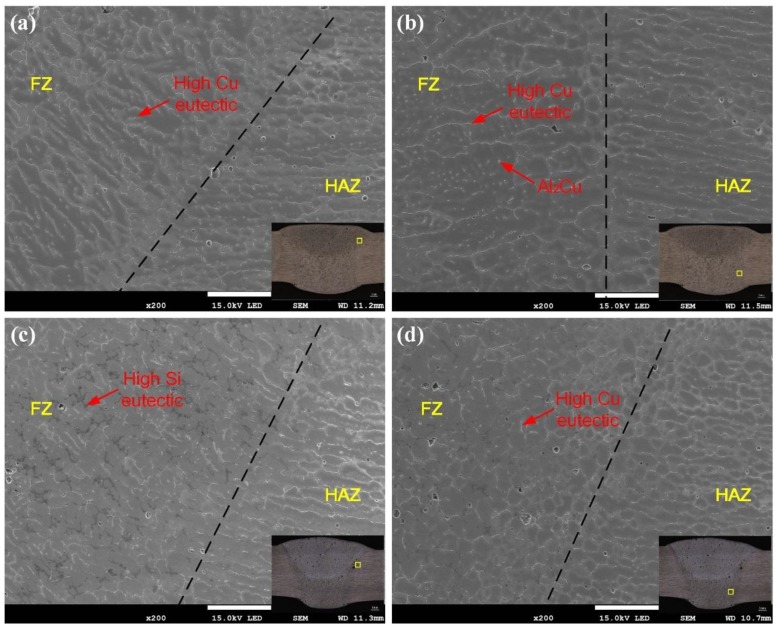
Fusion line microstructures of 2195-T6 welding joints using Al-Cu (**a**, **b**) and Al-Si-Cu (**c**, **d**) filler wires.

The microstructures of the fusion line side are depicted in Figure 6. The grain of the top layer was grown in the form of columnar crystal along the boundary of the fusion line. The grain boundary contained a large amount of copper-rich phase, the grain in the bottom layer was obviously enlarged, and the grain contained a dispersed second phase. In addition, it can be seen from Figure 6c,d that the crystal grains along the two sides of the fusion line exhibited a large difference, since the welding wire contained more Si and Cu element, and copper-rich and silicon-rich phases were distributed on the grain boundaries of the fusion zone. When using the Al-Si-Cu wire, it was seen that the grain of the bottom layer or the cover layer was significantly reduced, because more fine-grain element Ti was added to the wire. During the cooling process, the Ti element provided more nucleation locations and reduced the energy required for nucleation.

### 3.4. Distribution of Alloying Elements

**Figure 7:**
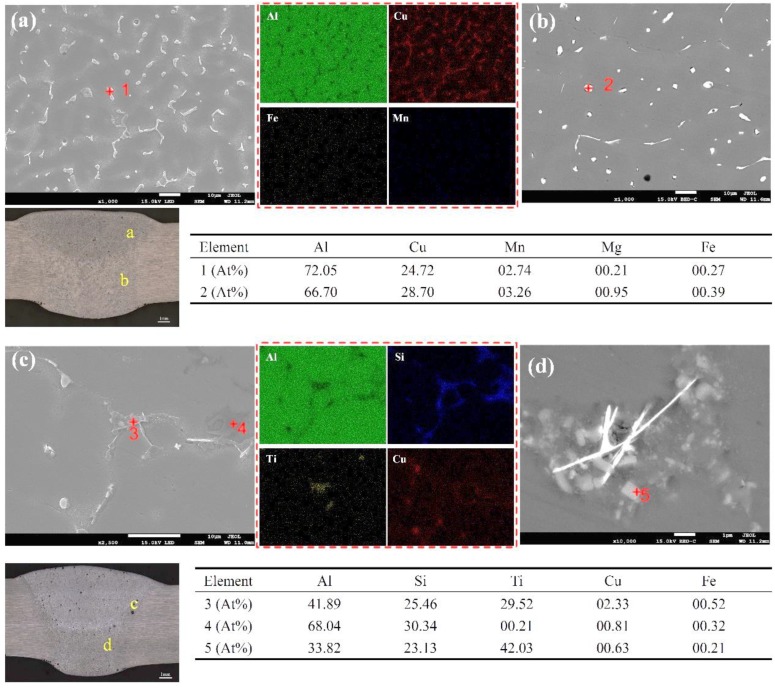
Distribution of alloying elements of 2195-T6 welding joints using Al-Cu (**a**, **b**) and Al-Si-Cu (**c**, **d**) filler wires.

The element distribution and chemical composition analysis of 2195-T6 welding joints with Al-Cu and Al-Si-Cu filler wires are displayed in Figure 7. More Cu elements were distributed on the grain boundaries and inner grains, as shown in Figure 7a,b. The Cu elements existed in the form of Al/Cu eutectic on the grain boundaries; however, the Cu content increased slightly inside the grain. Consequently, according to the EDS data and the phase diagram, it could be found that the substance with a dispersive distribution in the grains was the strengthening second phase Al2Cu. Additionally, more Si, Cu, and Ti elements were found in the welding joint with Al-Si-Cu filler wires, as shown in Figure 7c. The Si and Ti elements were mainly concentrated on the grain boundaries, while the Cu elements were not only distributed at the grain boundaries, but also within the grains. The EDS results indicated that there were many intermetallic compounds composed of Al, Si, and Ti at the grain boundaries, and some studies have shown that AlSiTi is a kind of ternary ceramic phase material [21,22,23]. When the grain boundary was magnified and detected by EDS, as shown in Figure 7d, it could be seen that there were substances exhibiting a regular rectangular shape and a high Ti content on the grain boundary. In addition, according to Figure 7c,d, it was seen that Si elements were widely distributed at the grain boundaries in the form of eutectic, which increased the brittleness of the grain boundaries. In conclusion, more brittle materials were distributed at grain boundaries in the welding joint using Al-Si-Cu filler wires, while less brittle material (Al_2_Cu) was found at the grain boundaries in the welding joint using Al-Cu filler wires.

### 3.5. Microhardness Analysis 

**Figure 8:**
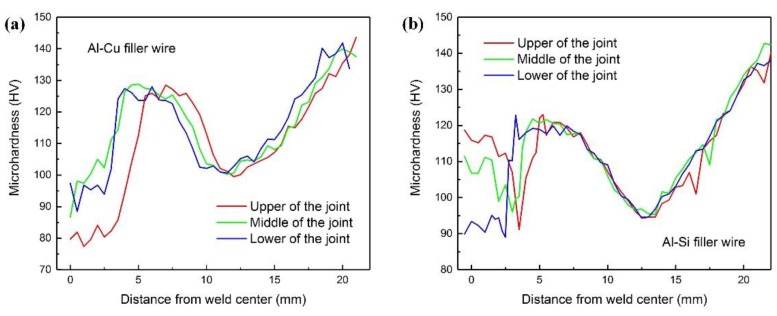
Microhardness distribution of the welding joints using Al-Cu (**a**) and Al-Si-Cu (**b**) filler wires.

Figure 8 shows the microhardness distribution of the welding joints with Al-Cu and Al-Si-Cu filler wires, respectively. There are three differences in the hardness distribution. 

First of all, the microhardness of the fusion zone (FZ) in Figure 8a is lower than that in Figure 8b. This is mainly attributed to the high content of Si and Ti in the filler wires, which forms hard and brittle intermetallic compounds containing Al/Si/Ti when the filler metal transits to the weld. Second, the microhardness of the two sides of the fusion line gradually increased when using the Al-Cu filler wire, but when the Al-Si-Cu filler wire was used, the hardness decreased first and then suddenly increased. This is because the hardness of FZ is higher, and the elements of Si and Ti could not be quickly diffused to the base metal, as shown in Figure 7c,d. Consequently, the hardness value displayed a mutation on the side of the fusion line when using the Al-Si-Cu filler wire. Third, the minimum hardness value was seen at the upper joint when using Al-Cu filler wire, while the minimum hardness value was seen at the lower joint when using Al-Si-Cu filler wire. This may be attributed to the large heat input of the top layer in the joint with Al-Cu filler wire and the large heat input of the bottom layer in the joint with Al-Si-Cu filler wire. The larger the heat input, the less numerous the strengthening phases at the joint [24], which results in a decrease of the strength of the material.

### 3.6. Fracture Morphology Analysis 

**Figure 9:**
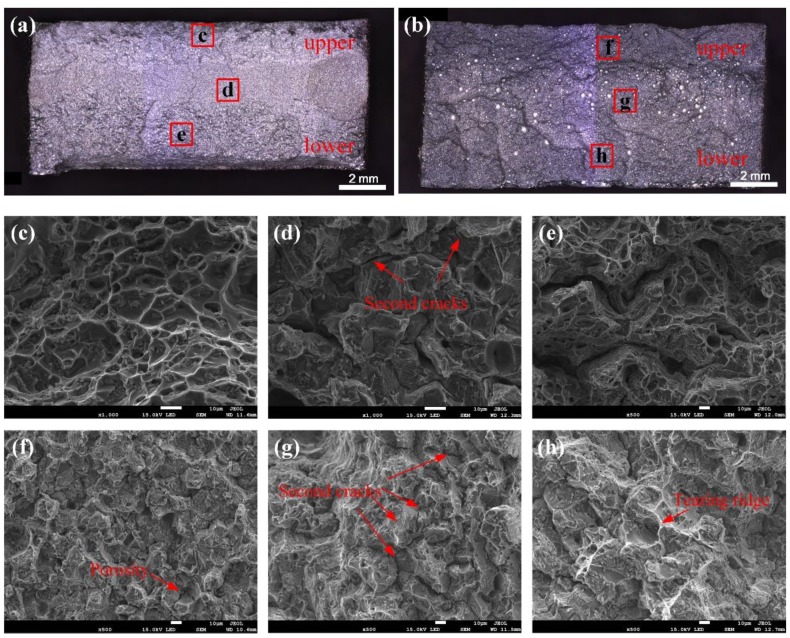
Fracture morphology of the welding joints using Al-Cu (**a**, **c**, **d**, **e**) and Al-Si-Cu (**b**, **f**, **g**, **h**) filler wires.

To better understand the failure difference of the welding joints, the fracture surface of the testing specimen was examined using SEM. Figure 9 shows the fracture morphology of the welded joints using Al-Cu and Al-Si-Cu filler wires. It can be seen that the fracture mode of the two welded joints are more different. First of all, the fracture morphology of the welded joint with Al-Cu filler wire can be divided into three parts: the top, the middle, and the bottom part along the thickness direction, as shown in Figure 9a. The top part is the fine grain zone with more dimples, while the middle part is the quasi-cleavage zone, which has some secondary cracks. There was more secondary phase inside the coarse grains of the bottom part; however, there were some cracks on the grain boundaries, as shown in Figure 9e. The fracture morphology of the welded joint with Al-Si-Cu filler wire was full of porosities (especially at the upper part in the middle of the joint) and exhibited a stepped pattern, as shown in Figure 9b. As can be seen from Figure 9f–h, the grain size of the upper part of the fracture was smaller than that of the lower part. More tear edges and secondary cracks indicated that the fracture mode of the welded joint with Al-Si-Cu filler wire was quasi-cleavage fracture.

**Figure 10:**
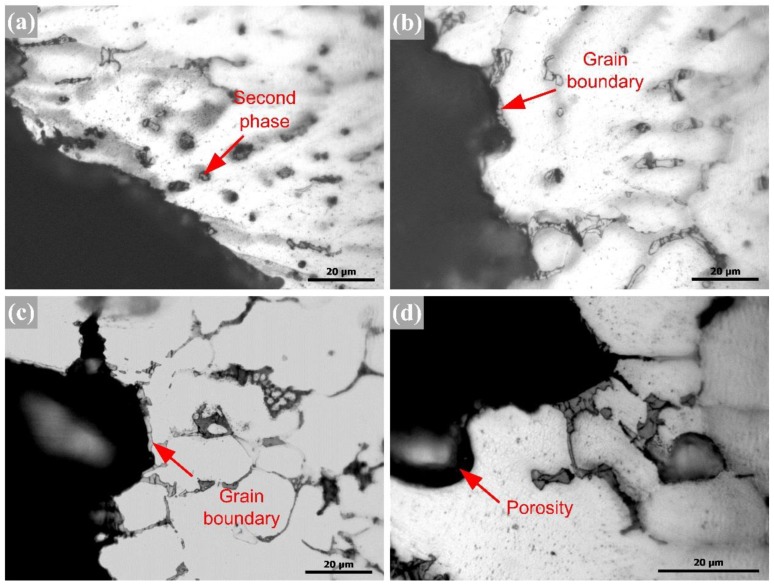
Fracture microstructures of the welded joints using Al-Cu (**a**, **b**) and Al-Si-Cu (**c**, **d**) filler wires.

Figure 10 shows the micro-morphology near the fractures. It can be seen that fracturing mainly occurred along eutectic phases on the grain boundaries and bypassed the strengthening phase inside the grains when using the Al-Cu filler wires. Additionally, grain boundaries and porosities were weak locations that were prone to stress concentration when using the Al-Si-Cu filler wires. 

**Figure 11:**
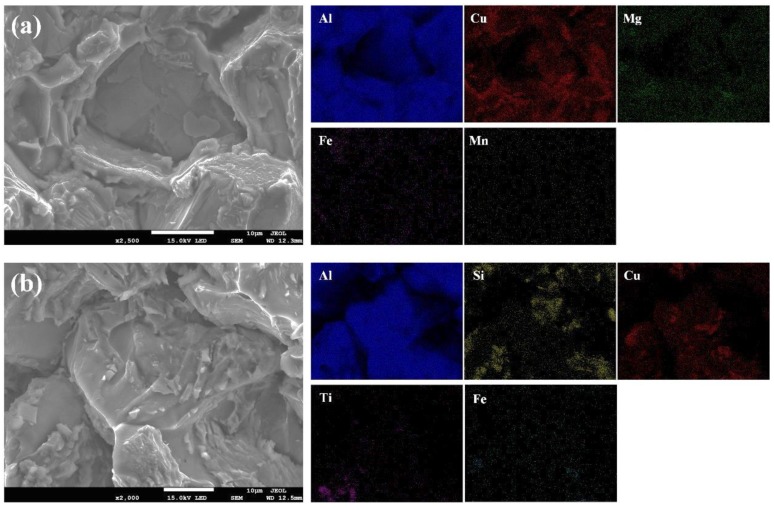
Distribution of alloying elements on the fracture surface when using Al-Cu (**a**) and Al-Si-Cu (**b**) filler wires.

In order to further verify the fracture mode of the specimen, an analysis of the element distribution on the fracture surface was carried out, and surface scanning was carried out at the middle position of the fracture surface. It was found that the main alloying element on the fracture surface (using Al-Cu filler wire) was Cu, which was mainly distributed on the tearing edge and grain boundaries, as shown in Figure 11a. However, the main alloying elements on the fracture surface were Si, Cu, and Ti, which were mainly distributed on the grain boundaries, as shown in Figure 11b.

## 4. Discussion 

During TIG welding, welding wires with different element contents are transferred to the weld metal, resulting in the redistribution of solutes, and the welded joints are formed after cooling. The equilibrium distribution coefficient ($k_{0}$) can be calculated as 0.171 and 0.132 in Al-Cu and Al-Si alloys, respectively, based on the phase diagrams. The degree of segregation can be described according to Equation (1) [25]:

(1) $$C_{s}-C_{l}=C_{\alpha }\left(1-k_{0}\right),$$

where $C_{s}$ and $C_{l}$ are the equilibrium concentration of solid and liquid, respectively, and $C_{\alpha }$ is the concentration of the main element, such as Cu and Si. It could be found that the smaller the $k_{0}$ ($k_{0}$ < 1), the larger the segregation trend. This was confirmed by the scanning of EDS, as shown in Figure 7. Furthermore, the grain boundaries mainly consisted of Al/Cu eutectic and some Al_2_Cu in the welded joint with Al-Cu filler wire, while the grain boundaries mainly consisted of Al/Si eutectic and some intermetallic compounds (Al_2_Cu/AlSiTi) in the welded joint with Al-Si-Cu filler wire. The Al-Si-Ti intermetallic compounds were brittle, with a lower ductility [22,26]. Furthermore, the welded joint had a non-uniformed microstructure, especially the side of the fusion line. 

Therefore, there are three aspects which can explain the fracture: (1) when a uniaxial load was applied to weld joints, the grain boundaries with higher strength phases hindered the movement of dislocation and caused the stress concentration; (2) the non-uniform distribution of the microstructure around the fusion line caused the microhardness to suddenly decrease; and (3) the porosities along the side of the fusion line reduced the effective bearing area of the sample and were also prone to stress concentration. 

**Figure 12:**
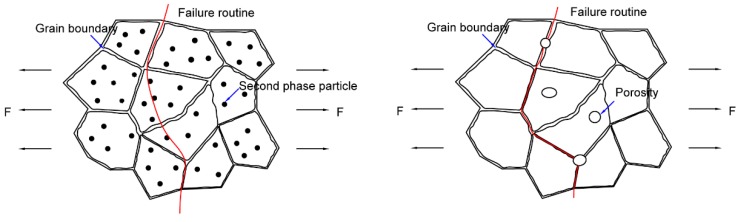
Schematic diagram of the failure routine on the welding joints when using Al-Cu (**a**) and Al-Si-Cu (**b**) filler wires.

By combining the analyses of fracture in Section 3.6, we can conclude that the fracture originates from the middle of the fusion line and the upper part of the bottom layer, where the fracture has a quasi-cleavage fracture mode and has an obvious secondary crack distribution. However, the top of Figure 9c and the bottom of Figure 9e, along the thickness direction of the fracture, there are many dimples, where the fracture has a ductile fracture mode. According to the former analysis, the main fracture routine of welding joints with Al-Cu filler wire is trans granular and occurs along the grain boundaries, as shown in Figure 12a. Under the action of uniaxial tension, the material surrounding the hard secondary phase first yields, while the secondary phase particles hinder the movement of dislocations. When the force was gradually increased, the stress concentration at the interface developed, which in turn caused crack generation. Subsequently, the cracks continued to extend and intersect along the weak position. Besides, regardless of the porosity content and the degree of segregation of the element, the Al-Si-Cu filler wire was always larger than the Al-Cu filler wire in the welded joint. The size and density of porosity in the welded joint with Al-Si-Cu filler wire were also bigger than in the welded joint with Al-Cu filler wire, as shown in Figure 9a,b. Hence, the effective bearing area of the specimen was seriously reduced and easily damaged in the position of a large pore when using Al-Si-Cu filler wire. The porosity and the grain boundary were the main weak locations, resulting in the emergence of cracks. A large amount of Si and Ti content intermetallic compounds were distributed in the grain boundary in the form of a net, and consequently, the grain boundary strength increased in the weld. However, these reinforcing phases were not present in the base metal, resulting in a large difference in the properties of the weld and the base metal, so the joint easily cracked along the fusion line under an external force. By combining the analyses of fracture, as shown in Figure 9b,f–h, it could be found that there was no dimple in the fracture, but there were secondary cracks and obvious tear ridges. Therefore, it can be concluded that the fracture is mainly a quasi-cleavage fracture. The element distribution and metallographic examination of the fracture surface show that the fracture was destroyed along the grain boundary and porosity, and the fracture routine can be represented by Figure 12b.

In conclusion, no matter the degree of element segregation or fracture process analysis, the joint strength and elongation when using Al-Si-Cu filler wire with high strength matching were not as good as those when using Al-Cu filler wire with low strength matching. 

## 5. Conclusions

In this study, the microstructure and mechanical properties of 2195-T6 Al-Li alloy spray formed by TIG welding with Al-Cu and Al-Si-Cu filler wires were studied. The following conclusions can be drawn:

1. The average strength and elongation of the TIG welding joint with Al-Cu filler wire were approximately 365 MPa and 6.29%, respectively, while those of the joint with Al-Si-Cu filler wire were approximately 302 MPa and 2.79%, respectively. In terms of the mechanical properties, the Al-Cu filler wire with low strength matching is suitable for welding space vehicle tanks;

2. The porosity area of the weld seam with the Al-Si-Cu filler wire was approximately 7.989 times larger than that of the Al-Cu filler wire, resulting in the joint property with Al-Si-Cu filler wire decreasing significantly;

3. The microstructure and microhardness results indicated that the Al/Cu eutectic near the fusion line distributed more at the grain boundaries, while more dispersed Al_2_Cu phase was located inside the grain, which improved the strength of the joint when using Al-Cu filler wire. However, when using the Al-Si-Cu filler wire, more Si, Cu, and Ti elements were segregated at the grain boundaries to form a brittle-hard network Al/Cu/Ti eutectic, which reduced the performance of the joint;

4. The fracture of the weld joint was a mixed fracture dominated by plastic fracture along the grain boundaries and bypassed the secondary phase particles when using Al-Cu filler wire. However, the fracture of the weld joint was mainly a quasi-cleavage fracture along the grain boundaries and the porosities when using Al-Si-Cu filler wire.

Overall, despite the high mechanical properties and ductility that were obtained in this study, there is still room for optimization to combat the existing problems of grain coarseness and cracks by adjusting the composition of filler wire.

## Figures and Tables

**Table 1:** Chemical compositions (wt.%) of filler wires.

Materials	Cu	Si	Ti	Zr	Fe	Cr	Mn	Al
Al-Cu filler wire	5.6	0.015	–	0.095	0.378	0.084	0.327	Bal.
Al-Si-Cu filler wire	2.18	5.5	1.53	–	0.42	0.103	–	Bal.

**Table 2:** Welding parameters of sprayed 2195-T6 alloy with Al-Cu and Al-Si-Cu filler wires.

Filler Wire	No. of Pass	Current (A)	Voltage (V)	Travel Speed (mm/min)	Shield Gas Flow Rate (L/min)		Heat Input (kJ/mm)
					Front	Rear	
Al-Cu	1 2	198 173	13.7~15.2 15.7~16.3	126.6 115.8	14	13	1.356 1.434
Al-Si-Cu	1 2	194 169	13.8~15.7 14.5~15.6	138.2 126.9	14	13	1.242 1.203

## References

[1] Rioja R.J. (1998). Fabrication methods to manufacture isotropic Al-Li alloys and products for space and aerospace applications. Mater. Sci. Eng. A.

[2] Ishchenko A.Y. (2005). High-strength aluminium alloys for welded structures in the aircraft industry. Weld. Int..

[3] Li J., Liu P., Chen Y., Zhang X., Zheng Z. (2015). Microstructure and mechanical properties of Mg, Ag and Zn multi-microalloyed Al–(3.2–3.8)Cu–(1.0–1.4)Li alloys. Trans. Nonferrous Met. Soc. China.

[4] Chen D.L., Chaturvedi M.C. (2001). Effects of Welding and Weld Heat-Affected Zone Simulation on the Microstructure and Mechanical Behavior of a 2195 Aluminum-Lithium Alloy. Metall. Mater. Trans. A.

[5] Abd El-Aty A., Xu Y., Guo X., Zhang S.H., Ma Y., Chen D. (2018). Strengthening mechanisms, deformation behavior, and anisotropic mechanical properties of Al-Li alloys: A review. J. Adv. Res..

[6] Hekmat-Ardakan A., Elgallad E.M., Ajersch F., Chen X.G. (2012). Microstructural evolution and mechanical properties of as-cast and T6-treated AA2195 DC cast alloy. Mater. Sci. Eng. A.

[7] Prasad N.E., Gokhale A., Wanhill R.J.H. (2013). Aluminum-Lithium Alloys: Processing, Properties, and Applications.

[8] Wang Y., Zhao G., Xu X., Chen X., Zhang W. (2018). Microstructures and mechanical properties of spray deposited 2195 Al-Cu-Li alloy through thermo-mechanical processing. Mater. Sci. Eng. A.

[9] Mazzer E.M., Afonso C.R.M., Galano M., Kiminami C.S., Bolfarini C. (2013). Microstructure evolution and mechanical properties of Al–Zn–Mg–Cu alloy reprocessed by spray-forming and heat treated at peak aged condition. J. Alloys Compd..

[10] Liu B., Lei Q., Xie L., Wang M., Li Z. (2016). Microstructure and mechanical properties of high product of strength and elongation Al-Zn-Mg-Cu-Zr alloys fabricated by spray deposition. Mater. Des..

[11] Godinho H.A., Beletati A.L.R., Giordano E.J., Bolfarini C. (2014). Microstructure and mechanical properties of a spray formed and extruded AA7050 recycled alloy. J. Alloys Compd..

[12] Wang S., Huang Y., Zhao L. (2018). Effects of different aging treatments on microstructures and mechanical properties of Al-Cu-Li alloy joints welded by electron beam welding. Chin. J. Aeronaut..

[13] Wang L., Suo Y., Wu C., Wang D., Liang Z. (2018). Effect of pulse frequency on microstructure and mechanical properties of 2198 Al-Li alloy joints obtained by ultrahigh-frequency pulse AC CMT welding. Materials.

[14] Zhang J., Feng X.S., Gao J.S., Huang H., Ma Z.Q., Guo L.J. (2018). Effects of welding parameters and post-heat treatment on mechanical properties of friction stir welded AA2195-T8 Al-Li alloy. J. Mater. Sci. Technol..

[15] Zhang X., Huang T., Yang W., Xiao R., Liu Z., Li L. (2016). A Microstructure and mechanical properties of laser beam-welded AA2060 Al-Li alloy. J. Mater. Process. Technol..

[16] Solórzano I.G., Darwish F.A., de Macedo M.C., de Menezes S.O. (2003). Effect of weld metal microstructure on the monotonic and cyclic mechanical behavior of tig welded 2091 Al–Li alloy joints. Mater. Sci. Eng. A.

[17] (2010). Metallic Materials–Tensile Testing–Part 1: Method of Test at Room Temperature.

[18] (2008). Tensile Test Method on Welded Joints.

[19] Dinnis C.M., Taylor J.A., Dahle A.K. (2006). Iron-related porosity in Al-Si-(Cu) foundry alloys. Mater. Sci. Eng. A.

[20] He E., Liu J., Lee J., Wang K., Politis D.J., Chen L., Wang L. (2018). Effect of porosities on tensile properties of laser-welded Al-Li alloy: An experimental and modelling study. Int. J. Adv. Manuf. Technol..

[21] Wu X.Y., Zhang H.R., Chen H.L., Jia L.N., Zhang H. (2017). Evolution of microstructure and mechanical properties of A356 aluminium alloy processed by hot spinning process. China Foundry.

[22] Singh V., Ghosh S., Rao P.V. (2012). Grindability improvement of AlSiTi conductive ceramic. Mater. Manuf. Process..

[23] Lei Y., Sun L., Ma W., Wei K., Morita K. (2016). Enhancing B removal from Si with small amounts of Ti in electromagnetic solidification refining with Al-Si alloy. J. Alloys Compd..

[24] Su C., Chen X., Gao C., Wang Y. (2019). Effect of heat input on microstructure and mechanical properties of Al-Mg alloys fabricated by WAAM. Appl. Surf. Sci..

[25] Ai C., Liu G., Liu L., Gong S., Zhang J., Fu H. (2012). Effects of Re and Ru additions on solidification partition coefficients and solidification characteristic temperatures of nickel base single crystal superalloys. Rare Met. Mater. Eng..

[26] Gao T., Li P., Li Y., Liu X. (2011). Influence of Si and Ti contents on the microstructure, microhardness and performance of TiAlSi intermetallics in Al-Si-Ti alloys. J. Alloys Compd..

